# Recent advances of natural biopolymeric culture scaffold: synthesis and modification

**DOI:** 10.1080/21655979.2021.2024322

**Published:** 2022-01-14

**Authors:** Jia Xin Yap, C.P. Leo, Nazlina Haiza Mohd Yasin, Pau Loke Show, Dinh-Toi Chu, Vijai Singh, C.J.C. Derek

**Affiliations:** a School of Chemical Engineering, Engineering Campus, Universiti Sains Malaysia, Nibong Tebal, Malaysia; b Department of Biological Sciences and Biotechnology, Faculty of Science and Technology, Universiti Kebangsaan Malaysia, Bangi, Malaysia; c Department of Chemical and Environmental Engineering, Faculty of Science and Engineering, University of Nottingham Malaysia, Semenyih, Malaysia; d Center for Biomedicine and Community Health, International School, Vietnam National University, Hanoi, Vietnam; e Department of Biosciences, School of Science, Indrashil University, Rajpur, Mehsana, India

**Keywords:** Biopolymer, cell culture, culture scaffold, collagen, chitosan, silk fibroin, gelatin, cellulosic, agarose

## Abstract

Traditionally existing 2D culture scaffold has been inappropriately validated due to the failure in generating the precise therapeutic response. Therefore, this leads to the fabrication of 3D culture scaffold resolving the limitations in the *in vivo* environment. In recent years, tissue engineering played an important role in the field of bio-medical engineering. Biopolymer material, a novel natural material with excellent properties of nontoxic and biodegradable merits can be served as culture scaffold. This review summarizes the modifications of natural biopolymeric culture scaffold with different crosslinkers and their application. In addition, this review provides the recent progress of natural biopolymeric culture scaffold mainly focusing on their properties, synthesizing and modification and application.

## 1. Introduction

Culture scaffolds are the platforms or support designed for cell growth or cellular interactions to form new functional tissues [1]. In the past decades, a wide range of cells has been cultivated on two-dimensional (2D) surfaces or scaffolds. Researchers have used these classical 2D cell culture systems to generate fundamental knowledge related to life science. However, the morphology of cells grown in 2D systems is significantly different from the morphology of cells in actual living tissues. Moreover, the flat 2D environment only allows the observation of cell growth through the top view. The analysis of gene expression, functional products, cell functions, and cell-to-cell interaction remains challenging [2].

The limitations mentioned above have sparked the creation of three dimensional (3D) cell culture scaffold for basic research, especially in biomedical sciences. The 3D cell culture is defined as a platform for cells to grow and reproduce, mimicking the natural environment [3]. 3D cultivation has been extensively adopted in recent years because of the development of bionic tissue models. The high-quality 3D models have been developed with tissue to recapitulate the biomimetic microenvironment observed under *in vivo* conditions. They are carefully refined to avoid the decompensation of the cellular signaling, division, and differentiation in traditional approaches of growing cells on the horizontal 2D culture planes. Cellular communication is limited within the circle with the trade-off in cell polarity, movement, and differentiation using these traditional approaches. The researches focus on the mechanisms controlling tissue or organ progress in tissue, and the studies have been extensively conducted using animal or human cells [4]. These research works conducted using in vitro 2D culture models can lead to misleading outcomes due to the formation of a monolayer product. Unlike their microenvironment in the animal or human bodies, the 2D cultured cells are not enclosed by supporting structures, including the extracellular matrix (ECM). Drug discovery studies also use cell-based bioassays to investigate the activity, metabolism, and toxicity of different drugs *in vivo*. The traditional techniques involving 2D cell culture are still widely adopted as they are simple and low in cost. Therefore, simple techniques and low-cost culture scaffolds allowing self-assembly cells into 3D designs and reproduction of cell interactions *in vitro* should be developed to advance tissue engineering, bioprinting, pharmaceutical products, cancer biology, and therapeutics [5].

Cells which for primary cell culture are obtained directly from an animal or plant tissue, while cells for secondary cell culture are obtained from an established primary culture. Therefore, secondary culture is a new culture originated from the primary culture. Primary cell cultures are important in manufacturing vaccines and therapeutic development. The global primary cell culture market was currently estimated at a size up to USD 3.4 billion. This market was predicted to grow at a compound annual growth rate (CAGR) of 11.6% in the next 7 years. Another recent market report shows that the cell culture market is projected to reach USD 33.1 billion by 2025. The market growth is primarily attributed to the increasing prevalence of chronic illnesses, such as cancer, infections, autoimmune diseases, diabetes mellitus, cardiovascular diseases, and nephrological diseases. In addition, the market growth is motivated by the growing need for cell culture-based vaccines, monoclonal antibodies (mAbs), experiments, and single-use products. All these factors have led to increased research, facilitating the high adoption of primary cell cultures. The primary cell culture systems from Thermo Fisher Scientific allow the researchers to closely mimic the cells *in vivo* state and create more physiologically relevant data. Furthermore, Merck Group offers primary human cells and optimized media for endothelial, epithelial, and fibroblast cell research. Lonza Group AG also provides cryopreserved and fresh primary cells to reduce the burden of self-isolation by the researchers [6,7].

There is a wide range of commercial scaffolds available in the current market as summarized in Table 1 [8–11]. In general, the materials of the available scaffolds are made up of cellulose, chitosan, gelatine, hydrogel, collagen, or biocompatible polymers, as listed in Table 1. Alvetex^TM^ 3D Cell Growth Plates manufactured by Thermo Scientific using synthetic polystyrene cost about $ 247.62 to $ 287.67 for a pack of 2 plates. Sigma produces cellusponge-collagen scaffolds, which cost about $ 1852.74 for a pack of 48 plates coated with inert hydroxypropyl cellulose and covered with collagen. Sigma also offers cheaper culture scaffolds, such as nanofiber cell culture dish, but it is still considered pricy as it costs $41.87 per plate. Another scaffold manufacturer, Corning produced a cell culture flask coated with fibronectin peptide and it is commercialized as PureCoatTM Amine 100 mm Dish with a price of $ 144.60 per case of 10.

**Table 1.** Examples of commercial 3D culture scaffolds

Commercial 3D Culture Scaffold	Price	Manufacturer	Main composition of scaffold	References
Alvetex™ 3D Cell Growth Plates	$ 247.62 – $ 287.67 (pack of 2 plates)	Thermo Scientific	Synthetic polystyrene scaffold.	[8]
Cellusponge-Collagen	$ 1852.74 (pack of 48 plates)	Sigma	Plates coated with inert hydroxypropyl cellulose covered with collagen.	[9]
Nanofiber cell culture dish	$ 41.87 (per plate)	Sigma	Nanofiber cell culture dish, with aligned nanofibers.	[10]
PureCoat™ Amine 100 mm Dish	$ 144.60 (per case of 10)	Corning	A cell culture flask coated with fibronectin peptide.	[11]

Looking into the recent reviews summarized in Table 2, there is a lack of critical review on the biopolymeric culture scaffold. Different types of culture scaffolds have been carefully discussed and compared, including synthetic polymers [12], graphene inks [13], paper [14], nanofiber [15,16], and hydrogel [17]. More strategies used in the preparation of culture scaffolds as well as their properties for bone tissue engineering and neural cell growth were explained [18]. Scaffold-free and scaffold-based approaches in cultivation and bioprinting besides exploring the challenges in scaffold fabrication [19], bioreactors designed for stem cell cultivation [20], biopolymeric scaffolds prepared through ice templating [21], the use of decellularized plant tissue, bacterial cellulose (BC), chitin, chitosan and recombinant collagen in cellular agriculture applications [22] were discussed.

**Table 2.** Recent reviews on culture scaffolds

Recent review papers	Authors and Year
Fabrication techniques of biomimetic scaffolds in three-dimensional cell culture: A review	[12]
Recent advances in ice templating: From biomimetic composites to cell culture scaffolds and tissue engineering	[21]
Colloidal systems toward 3D cell culture scaffolds	[18]
Scaffold-free 3-D cell sheet technique bridges the gap between 2-D cell culture and animal models	[19]
Scaffolds for 3D Cell Culture and Cellular Agriculture Applications Derived From Non-animal Sources	[22]
Graphene inks for the 3D printing of cell culture scaffolds and related molecular arrays	[13]
Bioreactor synergy with 3D scaffolds: New era for stem cells culture	[20]
Paper as a scaffold for cell cultures: Teaching an old material new tricks	[14]
Future of spermatogonial stem cell culture: Application of nanofiber scaffolds	[15]
Macroporous Hydrogel Scaffolds for Three-Dimensional Cell Culture and Tissue Engineering	[17]
Scaffolds for 3D in vitro culture of neural lineage cells	[16]

The major aim of this review is to analyze and compare the recent advances of natural biopolymeric culture scaffold on its synthesis and modification. With the help of biopolymer scaffolds, nutrients can be more evenly distributed to the cell attached on the scaffold. Moreover, 3D biopolymer scaffolds act as an extracellular matrix ECM in supporting the growth of cells. The cell culture can be structurally supported with the aid of natural biopolymeric culture scaffolds. However, it is still a challenge to harvest the cells for testing or subsequent sub-culture. The disadvantage of conventional cultivation, such as cell contamination which may spread quickly through the culture, limits the cells grown in such system. The biopolymer scaffold creates more specific physiological environments to improve the proliferation of cells [23,24]. More importantly, biopolymeric culture scaffolds offer higher sustainability and lower cost compared to other types of culture scaffolds.

## 2. Synthesis and modification of biopolymeric culture scaffold

### 2.1 Collagen culture scaffolds

Collagen, a fiber-like structure, is produced abundantly in the human body as it connects tissues together. Collagen is also one of the primary compounds in skin, tendons, muscles, bone, and cartilage as it provides elasticity and strength. Due to its biocompatibility, elasticity, and mechanical strength, collagen culture scaffolds have been widely developed through freezing and cross-linking, as summarized in Table 3.

**Table 3.** Collagen culture scaffold

Crosslinkers	Mechanical properties	Porosity	Pore size	Type of cultured cells	Medium used	Maximum growth rate	References
1-ethyl-3-(3-dimethylamino-propyl) carbodiimide (EDC) and N-hydroxysuccinimide (NHS)	6.71 ± 3.2 kPA and 6.73 ± 1.7 kPA for collagen-only and collagen- chondroitin sulfate scaffolds	N/A	9.48 ± 4.7 µm	Primary porcine trabecular meshwork cells	DMEM with 10% FBS and 1% pen/strep	Fluorescent imaging with 90% or more of the top surface of the collagen scaffold was covered with cells	[25]
EDC	N/A	N/A	N/A	Multipotent human mesenchymal stem cells (hMSCs)	Dulbecco’s modified Eagle’s medium (DMEM), α-minimum essential medium (α-MEM)	2.4 × 10^6^ cells/cm^3^	[26]
Gradient crosslinked	N/A	N/A	1.6 × 10^8^ pores/cm2	human small intestinal monolayers	expansion medium (EM)	Protein expression are more than 5 pmol/mg	[27]
N/A	Mechanical performance of the brain tissue: 6.9 kPa	N/A	N/A	Human neural stem cells, astrocytes, and microglia	N/A	cell viability of 84%	[30]
PFA, glutaraldehyde and osmium tetroxide 4 wt.%	Young’s modulus: 33 ± 12 kPa, Ultimate tensile Stress: 33 ± 6 kPa and Strain at failure: 105 ± 28%	N/A	25 to 95 µm	Normal Human Dermal Fibroblasts (NHDFs) and C2C12 murine myoblasts	low glucose Dulbecco’s modified Eagle’s medium (DMEM)	The cells show high proliferation rate under fluorescent microscopy	[31]
N/A	N/A	N/A	N/A	MLO-Y4 osteocytes	Minimum Essential Medium-α modification (α-MEM)	Fraction of dead cells of 0.0276 ± 0.0040	[33]
N/A	N/A	N/A	50 to 150 μm	human adipose-derived stem cells	DMEM	Quantification of differentiation rate of 75%	[29]
Glutaraldehyde	N/A	81 ± 3.9%	259.2 ± 45.2 µm	Hepatocyte (HepG2), liver cell cultivation	DMEM/high glucose	Cell proliferation with CCK-8 assay results of 2.7 at 450 nm absorbance, cell counting up to 5.8 × 10^5^ cells per piece	[32]
N/A	Young’s Modulus: 26 kPa	N/A	N/A	Adipose-derived mesenchymal stem cells (ADSCs) and osteoblasts	Dulbecco’s modified Eagle’s medium	Cell proliferation assay by CCK-8 analysis for 21 days: 800%	[34]
EDC/NHS	Tensile Modulus: 31 ± 4.4 MPa	92.18 ± 2.05%	138.06 ± 59.40 µm	Human umbilical vein endothelial cells	Endothelial Cell Growth Medium 2	Percentage of EdU-positive cells 24 h after seeding: 27%	[28]
N/A	Max elevation: 0.05 mm	N/A	N/A	Human dermal fibroblast cell	Dulbecco’s Modified Eagle Medium	Cell viability (LDH assay): 53 U/L	[35]

In short, collagen culture scaffolds are commonly synthesized through freeze-drying. They were applied in epithelial cells and adipose cells cultivation. The addition of crosslinkers such as EDC and NHS increased the stability of the scaffolds but weakened their mechanical strength. The higher the concentration of collagen in the scaffolds, the higher the porosity and the larger the pore sizes.

**Figure 1.**
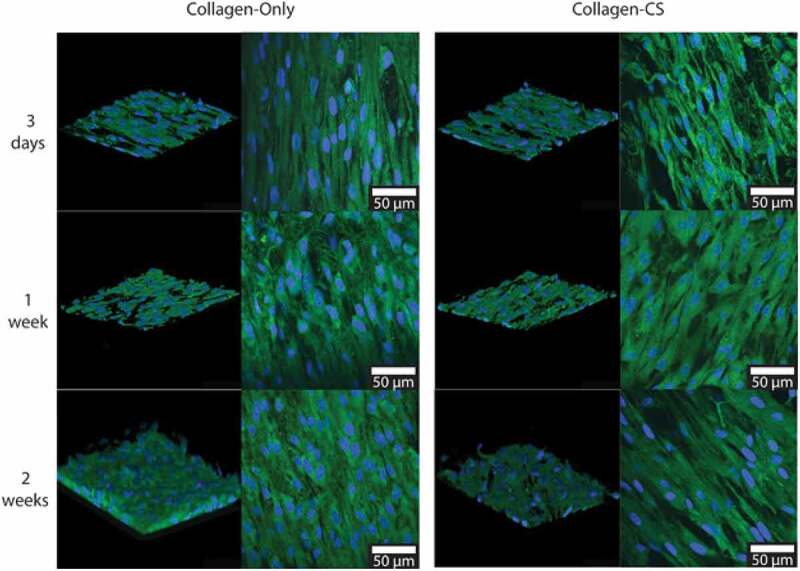
Fluorescent photomicrographs of trabecular meshwork cells seeded on the surface of collagen only and collagen-chondroitin sulfate scaffolds cells scaffold. The cells were labeled with DAPI (blue) and glutaraldehyde fixation (green). 100,000 cells were seeded on each scaffold. In the z-stack images, the top surface of the scaffold is at the bottom of the image. Scale bars represent 50 µm [25].

Culture scaffolds could be easily fabricated from collagen and collagen-chondroitin sulfate through unidirectional freezing and lyophilization, followed by dehydrothermal cross-linking for the cultivation of primary porcine trabecular meshwork cells [25]. Aligned pores formed with similar size and strength compared to the natural trabecular meshwork. Some collagen culture scaffolds were further cross-linked using 1-ethyl-3-(3-dimethylaminopropyl)carbodiimide (EDAC) and N-hydroxysuccinimide (NHS), but the retention of chondroitin sulfate in these scaffolds was not successfully increased. The elastic moduli near 6.70 kPA was recorded using collagen and collagen-chondroitin sulfate scaffolds without cross-linking. The trabecular meshwork cells on collagen-chondroitin sulfate scaffolds showed high viability as proven by the fluorescent images as shown in Figure 1. More than 90% of the top surface of the collagen scaffold was covered with cells. Proliferation and migration into the internal pore structure were observed two weeks after seeding. Besides that, porous scaffolds which made of jellyfish (*Rhopilema esculentum)* collagen were used for the cultivation of multipotent human mesenchymal stem cells (hMSCs) [26]. The collagen was fibrillated, which was later freeze-drying and cross-linking with N-(3-dimethylaminopropyl)-N’-ethylcarbodiimide hydrochloride (EDC). The researchers studied the effects of seed density, culture media with changing glucose loading and supplements such as fetal calf serum (FCS) and bovine serum albumin (BSA) on the chondrogenic differentiation. The ECM produced in this work was quantified in terms of sulfated glycosaminoglycan and collagen type II composition. The most robust upregulation of chondrogenic markers, along with the maximum ECM deposition was observed in scaffolds seeded with 2.4 × 10^6^ cells/cm^3^ and Dulbecco’s modified Eagle’s medium with great glucose content and 0.125% BSA. Collagen-coated porous membrane scaffold with EDC/NHS crosslinkers was applied in the cultivation of primary human small intestinal monolayers [27]. The small intestine monolayers on both porous membrane and collagen-coated porous membrane scaffolds exhibited goblet cells and abundant enterocytes. The cultured cells showed resistance to the permeation of Lucifer yellow and fluorescein-dextran (70 kD). Monolayers on the unmodified scaffold did not transport parzosin, the breast cancer resistance protein (BCRP). Cells on the modified collagen scaffold transported prazosin successfully, similar to the in vivo small intestines. Protein such as P-glycoprotein, BCRP, Na^+^/K^+^-*ATPase* and gamma-glutamyl transpeptidase expression more than 5 pmol/mg was recorded for the collagen modified scaffold. The drug transport involving the cell monolayer on culture scaffolds was affected by scaffolding stiffness. Furthermore, triple-helical peptides was introduced to improve cell-collagen scaffold interaction in the cultivation of human umbilical vein endothelial cells [28]. The collagen suspension was freeze-dried and then cross-linked using EDC to increase the stability of the collagen scaffold. The triple-helical peptides improved cells adhesion, cell spreading, and cell proliferation on 2D collagen films as well as 3D collagen culture scaffolds. The cross-linked collagen scaffolds with cross-linked appeared to be more stable, achieving cell death as low as 1.15 ± 0.08 at 490 nm. The cell density seeded on the modified collagen scaffolds increased up to 459.42 ± 28.58 cells per field of view. The increment could be related to the promiscuity of collagen-binding integrins for several collagen-binding proteins. Collagen sponge scaffold was prepared through freeze-drying and thermal cross-linking [29]. The cultivation and differentiation of human adipose-derived stem cells on collagen sponge scaffold were examined by indirect co-culture strategy. The collagen sponge scaffolds were prepared with chondroitin sulfate through freeze-drying, thermal cross-linking under vacuum at 105°C, immersion in glutaraldehyde solution, and cross-linking at 4°C. Those keratinocytes cultured on the collagen sponge scaffold exhibited more keratinocyte-like cells compared to those cultured in the air-liquid interface. Human adipose-derived stem cells adhered to the collagen scaffold successfully, and the confluency reached more than 90% that cultured in complete Dulbecco’s modified Eagle’s medium (DMEM). Cell proliferation was observed inside the pores along the pore walls after 15 days of cultivation, indicating good cell adhesion and proliferation. Hematoxylin and eosin (HE) staining also revealed that human adipose-derived stem cells co-cultured over the collagen scaffold expressed K14 at higher levels, and the differentiation rate reached about 75% at day 15.

In addition, collagen culture scaffolds were produced without cross-linking. Biopolymer scaffolds which consisted of the concentration of collagen I of 3.0 mg/mL, hyaluronic acid in the concentration range of 1.0 to 2.0, and poly(ethylene glycol) diacrylate in the concentration of 7.0 to 8.0 were fabricated and characterized to be used for the cultivation of human neural stem cells, astrocytes, and microglia [30]. The fabricated collagen scaffolds were able to maintain the complex moduli of the mechanical performance of the brain tissue within the range of 6.9 kPa. Moreover, the cell viability of the cell cultivation up to 84% was achieved. However, the viability of human neural stem cells was the lowest as the sensitive stem cells need specifically designed scaffolds and conditions to grow. The topotactic transformation of collagen into macroporous monoliths through freeze-drying has been reported [31]. The wet collagen scaffolds without cross-linking showed noble mechanical characteristics, including Young’s modulus of 33 ± 12 kPa. The ultimate tensile stress of 33 ± 6 kPa and strain at failure of 105 ± 28% were also recorded. The collagen scaffolds were further tested in the cultivation of normal human dermal fibroblasts (NHDFs) and C2C12 murine myoblasts. The results indicated that the cultured cells successfully migrated, and they formed well-oriented populations in the 3D biomimetic collagen scaffolds. Collagen I was impregnated into polyvinyl alcohol (PVA) scaffold after modification with glutaraldehyde to create a highly hydrophilic and biologically active scaffold for the cultivation of liver cells, hepatocyte (HepG2) [32]. The composite collagen culture scaffold with the highest collagen content of 12.13% showed the highest porosity of 81 ± 3.9% with interconnected pores of pore size 259.2 ± 45.2 µm. The collagen-modified PVA scaffolds exhibited an increment in cell proliferation with CCK-8 assay results of 2.7 at 450 nm absorbance and cell counting up to 5.8 × 10^5^ cells per piece. In addition, albumin secretion reached 4.2 mg/10^6^ cells, and urea synthesis with a fold increased of about 1.6 at day 15 using the collagen modified PVA scaffolds compared to those without collagen.

Besides the flat culture scaffolds, collagen culture scaffolds are available in the form of beads. A rotary cell culture system was developed using collagen-hydroxyapatite bead scaffolds for the cultivation of MLO-Y4 osteocytes [33]. A mean stiffness value of 0.909 ± 0.047 kPa was obtained for the optimized scaffold produced from collagen-containing 6 mg/mL hydroxyapatite. The scaffold stiffness was significantly improved compared to the collagen scaffold without hydroxyapatite (0.515 ± 0.124 kPa). The cell viability of osteocytes was improved in collagen-hydroxyapatite scaffolds compared to collagen scaffolds without hydroxyapatite as well. The fraction of dead cells was 0.0276 ± 0.0040, 3 times lower than the latter. Collagen-hydroxyapatite scaffolds further promoted osteocyte-specific gene expression. Paracrine effect of adipose-derived mesenchymal stem cells (ADSCs) and osteoblasts cultivated using collagen modified alginate microparticles was studied [34]. The alginate microparticles containing osteoblast were prepared during electrostatic droplet extrusion. The encapsulation of osteoblasts was manipulated by adjusting the voltage supplied using electrostatic droplet extrusion. Then, the alginate microparticles were impregnated with collagen. The osteogenesis of stem cells was improved with cell proliferation as proven by cell counting kit-8 assay (CCK-8) analysis, up to 400% at day 21. Osteoblasts subsequently induced osteogenic differentiation of human adipose-derived stem cells (ADSCs) without any exogenous factors, indicating a potential scaffold system in stem cell therapy for bone regeneration. Moreover, co-cultured ADSC and osteoblasts exhibited more bone creation in comparison to the ADSC monoculture group of the rat calvarial defect model. Human dermal fibroblast cells were cultured on the porous collagen scaffold [35]. They further designed a perfusable system that provided nutrient diffusion and oxygen convection to the cultured cells. The viability of cells was greatly improved, achieving a lactate dehydrogenase (LDH) concentration of 42 U/L. The necrosis of cells was successfully prevented using the porous collagen scaffold. With the perfusable channels, glucose consumption and lactate production of fibroblast cells was increased up to 405 mg/mL and 33 mg/mL, respectively. The mechanical stress of the perfusable system with the collagen culture scaffold showed no damage to the cultured cells.

### 2.2 Chitosan culture scaffolds

Chitosan extracted from shells and exoskeleton is a linear polysaccharide with biocompatibility and biodegradability are desired in developing culture scaffolds. This chelating agent interacts with protein, fat, cholesterol, and ions satisfactorily. Hence, various chitosan culture scaffolds have been developed, as listed in Table 4.

**Table 4.** Chitosan Culture Scaffold

Crosslinkers	Mechanical properties	Porosity	Swelling degree	Type of cultured cells	Medium used	Maximum growth rate	References
N/A	Tensile strength: 1.33 ± 0.26 MPa	86.69 ± 1.97%	N/A	dental pulp stem cells	minimum essential medium Eagle	Cell number: 5 × 10^5^ cells/scaffold	[36]
N/A	Young’s modulus of 27.7 kPa for 8% chitosan-hyaluronic acid	>90%	N/A	human glioblastoma cells (U-87 MG)	minimum essential medium	Cell number: 2.0 × 10^5^ at day 9	[37]
Form of hydrogel	N/A	N/A	N/A	Bone marrow-derived mesenchymal stem cells	High glucose Dulbecco’s modi fied eagle’s medium	N/A	[38]
Form of hydrogel	Tensile strength: 4.1 MPa	N/A	50%	Mesenchymal stem cells	AmnioGrow, DMEM and KSFM	N/A	[40]
1-ethyl-3-(3-dimethylamino-propyl)carbodiimide (EDC) and N-hydroxysuccinimide (NHS)	N/A	N/A	N/A	Hepatocyte (Human liver cells)	Dulbecco’s modified Eagle’s medium (Hight Glucose)	Cell proliferation (CCK8 assay at 450 nm): 2.3	[39]
N/A	Stress at 50% compression: 12 kPa	68%	N/A	Osteoblast/Osteoclast (Bone cells)	alpha minimum essential medium	3.2 × 10^5^ cells per scaffold	[41]

Chitosan is needed to dissolve in acetic acid before being fabricated into culture scaffolds. Additives such as peptides and calcium phosphate were added with chitosan in the scaffold preparation to increase the cell adhesion and proliferation while enhancing the mechanical strength of scaffolds. Mesenchymal stem cells were mainly used to cultivate on chitosan culture scaffold.

**Figure 2.**
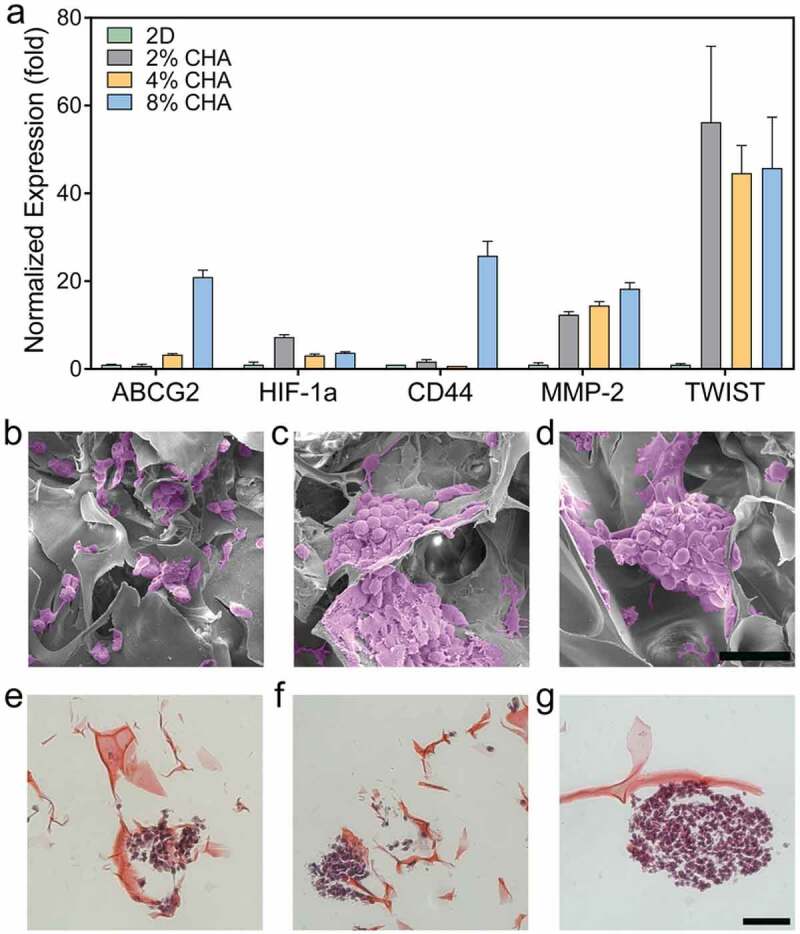
Gene expression and morphology of cells cultured on different substrates. (a) Relative expression displaying increased drug resistance and invasion specific genes in 3D scaffolds by U87-MG RFP cells after 12 days of culture (n = 3). Error bars represent standard error of mean. Scanning electron micrographs of GBM cells in (b) 2% Chitosan and hyaluronic acid (CHA), (c) 4% CHA, and (d) 8% CHA where tumor spheroid size increases with increasing stiffness. Histological staining (H&E) of tumor spheroids on (e) 2% CHA, (f) 4% CHA, and (g) 8% CHA scaffolds. Scale bars represent 100 μm (b-d) and 50 μm (e-g) [37].

Different types of additives were used in the preparation of chitosan culture scaffolds. A porous chitosan-xanthan matrix scaffold was developed to study the cultivation of dental pulp stem cells [36]. Chitosan in acetic acid solution was reacted with xanthan containing surfactant (Poloxamer), followed by drying and washing. To promote the differentiation of stem cells into chondrocytes, they further added kartogenin. They produced an amorphous scaffold with an average thickness of 0.89 ± 0.01 mm and a great absorptivity up to 13.20 ± 1.88 g culture medium/g. The kartogenin content down to 100 nmol/L was adequate to promote differentiation into chondrocytes, showing the effectiveness of this strategy for the treatment of cartilage lesions in tissue engineering. Besides, chitosan-hyaluronic acid polyelectrolyte complex scaffolds with varied stiffness were fabricated [37]. Chitosan and hyaluronic acid were dissolved separately in acetic acid before mixing, freezing (12 h at 4°C and 24 h at −20°C), and freeze-drying. The effects of scaffold stiffness on scaffold morphology, proliferation rate, and drug resistance were studied. Moreover, the gene expression in human glioblastoma cells (U-87 MG) was examined. The results of gene expression and morphology of cells are shown in Figure 2. All scaffolds supported glioblastoma multiforme proliferation throughout 12 days of cultivation. However, larger spheroids were observed in the scaffolds with higher stiffness. Glioblastoma multiforme cells cultured in the chitosan-hyaluronic acid scaffolds with high stiffness attained more invasion-related genes and resistance against the usual chemotherapeutic temozolomide. The increasing drug efflux resulted in increased cell survival. Hydroxybutyl chitosan hydrogel with Arg-Gly-Asp peptide was modified to improve the adhesion and proliferation of mesenchymal stem cells originated from bone marrow within the hydrogel [38]. The peptide was dissolved in 2-morpholino-ethanesulfonic acid buffer and then added with EDC/NHS crosslinkers. The mixture was subsequently mixed with hydroxybutyl chitosan solution before lyophilization. The modified hydroxybutyl chitosan hydrogel with Arg-Gly-Asp was more beneficial than hydroxybutyl chitosan hydrogel in cell adhesion and proliferation. The mesenchymal stem cells incorporated in the modified hydroxybutyl chitosan hydrogel with Arg-Gly-Asp obstructed the proliferation of keloid fibroblasts which are the tumor cells. Since the chitosan hydrogel could suppress the nodular collagenous fibers of keloid tissue, it is considered a potential candidate in keloid therapy. Porcine liver ECM coatings on chitosan fabrics were synthesized as the culture scaffold for hepatocyte, human liver cells [39]. Porcine liver was decellularized using 1% Triton X-100 before coating on the chitosan scaffold. The porcine liver ECM attached covalently to the chitosan scaffold using EDC/NHS crosslinkers. The hepatocytes growing on the coated chitosan scaffolds showed encouraging cell bioactivity and liver-specific activities. The synthesis of albumin secretion and urea were detected around 270 µg/mg total protein per 24 h and 340 µg/mg total protein per 24 h at day 7, respectively.

Inorganic additives were also incorporated into chitosan culture scaffolds. Chitosan scaffolds doped with gold (Au) nanoparticles were fabricated for mesenchymal stem cell culture associated with microwave-assisted synthesis [40]. The scaffolds were fabricated with a deacetylation degree in the range of 80–100%, irradiation time of 30 min, temperature range of 160–180°C, between 200 to 300 W in 40% NaOH solution. Aspartic acid and glycerol crosslinker were also added. The cross-linked scaffold without Au nanoparticles showed the highest swelling degree of 50%. The cross-linked scaffold with Au nanoparticles exhibited a uniform structure and high porosity, which enabled nutrients, gases, and metabolites diffusion for cells divisions and maturation. The chitosan scaffold with 100% of deacetylation degree and 0.2% of Au nanoparticles showed the maximum tensile strength of 4.1 MPa and the optimum rigidity with elongation at break of 41%. Furthermore, this scaffold also exhibited antibacterial activity with an increased inhibition zone of 33–35 mm. Porous chitosan scaffolds with calcium phosphates were modified, followed by hemocyanin coating to cultivate human bone marrow stromal cells (hBMSCs) [41]. The chitosan scaffolds were fabricated with a deacetylation degree of 90% and a viscosity of 50 mPa·s through freeze-drying and NaOH stabilization before modification. The addition of calcium phosphates caused an increase in scaffold bioactivity by increasing the strength of chitosan scaffolds. Hence, the calcium concentration in the culture medium, the differentiation hBMSCs, and osteoclasts maturation in mono-culture were reduced. On the contrary, the modification of chitosan scaffolds by dip-coating with hemocyanin proteins showed a significantly increased proliferation of hBMSCs and equivalent osteoclast maturation. For the co-culture, mineral incorporation exhibited an inhibitory effect on osteoblastogenesis, whereas a stimulatory effect was observed in hemocyanin-modified scaffolds.

### 2.3 Silk fibrion culture scaffolds

Silk fibroin is an amphiphilic block copolymer obtained from Bombyx mori silk. This natural fibrous protein is biocompatible for different biomedical applications. Silk fibroin culture scaffolds that have been recently developed are summarized in Table 5.

**Table 5.** Silk fibroin culture scaffold

Crosslinkers	Mechanical properties	Porosity	Pore size	Swelling degree	Type of cultured cells	Medium used	Maximum growth rate	References
N/A	Interfacial strength: 57.3 ± 0.9 kPa	N/A	N/A	N/A	osteoblast and macrophage	Dulbecco’s modified Eagle’s medium	Bone formation: 70%	[42]
N/A	N/A	71 ± 2%	130 ± 23 μm	95.8 ± 1%	apical papilla stem cells	Dulbecco’s modified Eagle’s medium	DNA concentration: 120 ng/µl	[44]
N/A	N/A	42.06 ± 4.83%	100 µm to approximately 200 µm	N/A	Osteoblast	Dulbecco’s modified Eagle’s medium	Cell adhesion rate: 95%	[49]
N/A	Young’s modulus: 0.5 ± 0.6 MPa	N/A	N/A	N/A	bone marrow mesenchymal stem cells	Dulbecco’s modified Eagle’s medium	Cell viability: OD405 value 19.289 to 35.174	[43]
N/A	N/A	N/A	N/A	N/A	mesenchymal stem cells	Dulbecco’s modified Eagle’s medium	DNA quantification: 0.6 sGAG/DNA	[45]
N/A	N/A	82.2 ± 1.3%	3 to 212 µm	255%	mesenchymal stem cells	Dulbecco’s modified Eagle’s medium	Cell density: 900 cells/mm^2^	[46]
N/A	N/A	86 to 90%	100.93 ± 1.36 μm	N/A	Human mesenchymal stem cells	Dulbecco’s modified Eagle’s medium	Cell distribution: 550 ± 42 cell/mm^2^	[47]
N/A	N/A	>96%	150–170 µm	N/A	Articular chondrocytes and adipose-derived human mesenchymal stem cells	Dulbecco’s modified Eagle’s medium	DNA content: 7500 ng/g of scaffold	[48]
PAMAM dendrimers (0.5% EDC and 1% NHS)	N/A	N/A	N/A	N/A	L929 fibroblast cells	McCoy’s 5 A medium	Cell viability: 110%	[4]
N/A	Stress: 680 kPa	88.8%	300 um	758.9%	Stem cells from human exfoliated deciduous teeth (SHED)	α-modified Eagle’s medium	Albumin secreted: 1.4 g/dL	[50]

Silk fibroin scaffolds were frequently incorporated with chitosan to increase the scaffold hydrophilicity, porosity, and pore size during ice-templating in the past. The thermal stability and mechanical strength of silk fibroin scaffolds could be further enhanced by adding reduced graphene and chondroitin. The silk fibroin scaffolds were successfully applied in the cultivation of mesenchymal stem cells.

Silk fibroin culture scaffolds could be produced through electrospinning. In vitro osteogenic differentiation of hMSCs and in vivo bone tissue regeneration were studied using the mineralized nanofibrous polymeric scaffolds [42]. Hydroxyapatite was deposited on the non-mulberry silk fibroin functionalized poly(epsilon-caprolactone) nanofibrous scaffolds through electrodeposition, co-electrospinning, or alternative soaking. Bone morphogenic protein-2 and transforming growth factor-β were covalently grafted to the mineralized nanofibrous scaffolds at a potency ratio of 1:1. Both i*n vitro* and *in vivo* osteogenesis and osseointegration studies disclosed the greatest bone formation on the scaffolds mineralized through electrodeposition, up to 70% followed by alternate soaking and co-electrospinning. All scaffolds attained reduced immunoreactivity with the maximum formation of tissue, about 91.5%. Electrospun silk fibroin culture scaffold containing reduced graphene oxide (0.5%, 1.0% and 2.0% (w/v)) were developed [43]. The scaffolds were immersed into methanol for 45 min to initiate conversion into β-sheet after electrospinning. The presence of reduced graphene oxide strengthened the intermolecular forces between reduced graphene oxide and silk fibroin molecular chains, resulting in a rise in silk II content. Besides that, the reduced graphene oxide enhanced the thermal and mechanical stability of the silk fibroin scaffolds. The silk fibroin scaffold containing the reduced graphene oxide supported cell viability with OD405 value ranging from 19.289 to 35.174. Bone marrow mesenchymal stem cells were prompted osteogenically for 30 days, showing that the modified silk fibroin scaffold supported osteogenic differentiation.

**Figure 3.**
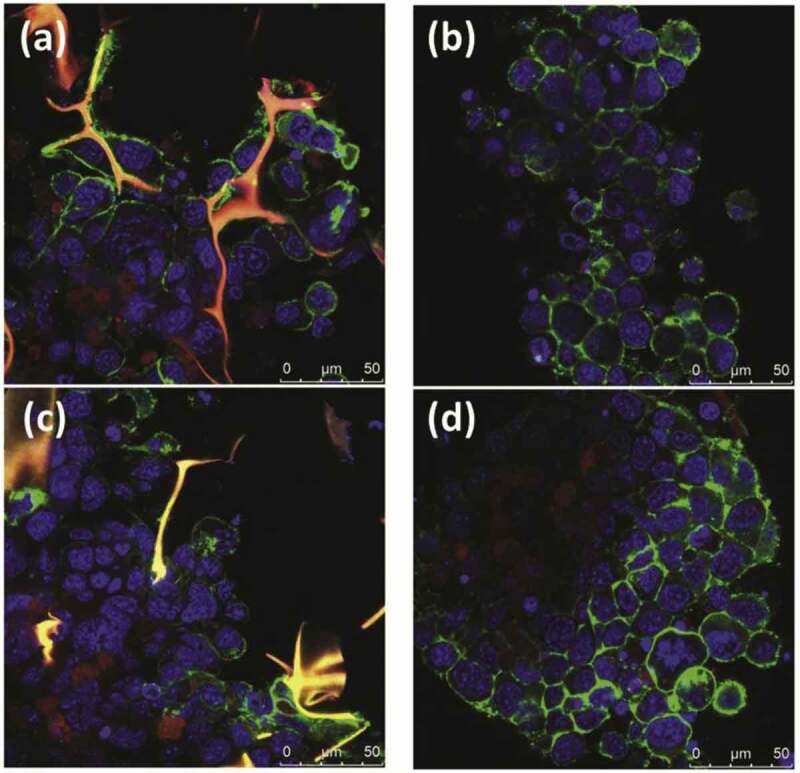
Confocal microscopy images of chondrogenic differentiated hMSCs on (a and c) SF/CS-Gl-Ch scaffolds and (b and d) pellet culture, where red represents immunofluorescence of Col II in a-b and Acan in c-d. Green fluorescence represents β-actin stained by phalloidin and blue corresponds to nucleus stained by Hoechst 33258 [47].

However, ice templating has become more prevalent in recent works focusing on silk fibroin culture scaffolds. Silk fibroin scaffolds were prepared through ice templating at −80°C for 12 h, followed by freeze-drying and incubation of culture medium before seeding of apical papilla stem cells [44]. They further investigated the effects of pre-incubation time on cell attachment and proliferation. The roughness and water contact angle could be reduced by increasing pre-incubation time from 2 d to 10 d. The DNA measurement after cell seeding displayed a noteworthy increment of DNA content up to 120 ng/µl after 10 d. More protein was absorbed since the hydrophilicity of scaffolds increased over time. In vitro cartilage construction on silk fibroin/chitosan composite scaffold was studied under dynamic culture conditions [45]. The silk-fibroin/chitosan scaffolds at a volume ratio of 80:20 were prepared through ice templating at −80°C for 24 h. The hMSCs from umbilical cord blood were cultured on the silk-fibroin/chitosan composite scaffolds. The cell density increased up to 543% in a spinner flask with 62% live cells, higher than the cells under static culture conditions. The DNA and glycosaminoglycans content after 3 weeks indicated that the chondrogenic differentiation was improved under the dynamic culture conditions. Human articular cartilage with a thickness of 5 mm was successfully constructed. The effects of chondroitin sulfate on the morphology and functionality of silk fibroin/chitosan blend scaffolds were further investigated [46]. The scaffolds were prepared through ice-templating and freeze-drying. The addition of chondroitin sulfate caused a rise in porosity and surface hydrophilicity. The water contact angle on scaffolds reduced from 54.2 ° to 46.8 °. The polymerase chain reaction data affirmed the chondrogenic differentiation in terms of increasing expression of collagen-II, Sox9, and aggrecan but reducing expression of collagen-I. In their subsequent work, silk fibroin/chitosan scaffolds containing cartilage matrix compounds such as glucosamine and chondroitin sulfate were prepared for the cultivation of umbilical cord blood-derived hMSCs [47]. Silk fibroin dissolved in water and chondroitin sulfate dissolved in acetic acid were mixed at a volume ratio of 80:20 before adding glucosamine and chitosan (0.5–1.5% w/v). The mixture was freeze-dried at −80°C for 1 d and −100°C for 1 d. The silk fibroin/chitosan scaffolds with cartilage matrix compounds showed a pore size distribution between 56.55 and 168.15 μm, a high porosity up to 92%, water contact angles as low as 44.7 °, and restricted swelling. DNA content increased two times after 14 d, while the highest cell number was achieved after 21 d using the silk fibroin/chitosan scaffolds with 1.5% chitosan in a spinner flask bioreactor. The improvement of optical density (OD) on the scaffolds was confirmed using 2-(4,5-Dimethylthiazol-2-yl)-2,5-diphenyltetrazoliumbromide (MTT), indicating the fast adaptation of cell to the porous scaffolds. The production of the chondrogenic ECM and gene was proven in the histology, immunophenotype, immunofluorescence as shown in Figure 3 and qPCR analysis due to the homogenous distribution of nutrients within the porous scaffold and the stimulation by the cartilage matrix compounds. On the other hand, the effects of cellular interaction in the co-cultivation of articular chondrocytes and adipose-derived hMSCs on silk fibroin scaffolds were studied [48]. They produced the scaffolds by freezing silk fibroin solution (2%) at −20°C, then freeze-drying for 1 d. They further established co-culture model was developed using different ratios of articular chondrocytes to adipose-derived hMSCs. A higher proliferation and cellular viability with the DNA content up to 7500 ng/g was recorded compared to the monoculture groups. Co-culture models exposed the synergistic connection among the cells with interaction index values falling between 2 to 3.

Several works reported on the silk fibroin culture scaffold incorporated with other cross-linked polymers. Scaffolds produced from silk fibroin with chitosan and/or gelatin were used for the cultivation of mice preosteoblasts, MC3T3-E1 cells [49]. The mixture of biopolymer solution was cross-linked using EDC/NHS in ethanol solution before freezing at −20°C for 1 d and −80°C for 1 d. The largest pore size was recorded for the silk fibroin/chitosan/gelatin scaffold, ranging from 100 µm to 200 µm for effective cell infiltration. However, these scaffolds exhibited a low porosity of about 42% with a low water absorption rate of 537.34 ± 1.61%. The degradation of this scaffold, 32.11% after 10 d was ranked second after the silk fibroin scaffold. MTT assay of MC3T3-E1 cells cultured on the scaffolds showed the highest cell adhesion rate (90% at 1 h; 95% at 6 h), the highest cell proliferation rate with an optical density value of 0.75 recorded after 7 d. The surface of silk fibroin (SF) nanofibrous scaffold was modified by cross-linking PAMAM dendrimers to preserve the original structure of protein and provide a good affinity for cell attachment [4]. After electrospinning of silk fibroin in formic acid and crystallization in ethanol solution (75%), the dried silk scaffold was modified using EDC and NHS at 37°C for 120 min. Then, the scaffolds were immersed into 7% PAMAM in ethanol. The porous scaffold formed with an average diameter of 80 nm. The crosslinker concentration controlled at 0.5% of NHS and 1% of EDC resulted in satisfactory cross-linking between nanofibers. After seeding fibroblasts cell line L929 onto the fibroin scaffolds, MTT assay showed minimum cytotoxicity of the silk fibroin scaffolds. The cell viability reached 110%, slightly higher than the control group.

Silk fibroin culture scaffolds were also integrated into the dynamic system. Stem cells of human exfoliated deciduous teeth were cultured using the silk fibroin scaffolds with the NIH3T3 cultivated medium under static and dynamic conditions for 28 d [50]. The silk fibroin scaffolds were prepared by salt templating. NaCl (200–250 μm) was added to the hexafluoroisopropanol containing 10% (w/v) silk fibroin. The mixture was dried at room conditions for 3 d, followed by crystallization in methanol for 60 min, template removal in water for 1 d, and freeze-drying for 8 h. The porous scaffold supported hepatospheres formation and enhanced cell viability as shown by MTT assays. Under dynamic conditions, the use of scaffolds also improved hepatic gene expression, glycogen storage, albumin excretion, and urea production. The NIH3T3 cultivated medium induced hepatic differentiation of stem cells, while the 3D dynamic culture system enhanced the hepatic differentiation of cells.

### 2.4 Gelatin culture scaffolds

Gelatin is hydrolyzed collagen that is commonly used as the gelling agent. Table 6 shows the culture scaffolds made of gelatin. In brief, gelatine culture scaffolds were commonly used for the cultivation of epithelial cells and fibroblast cells. The porous structure of gelatine culture scaffolds promotes the diffusion of oxygen and nutrient between the scaffolds and the cells attached to scaffold surface to increase the cell viability.

**Table 6.** Gelatin culture scaffold

Crosslinkers	Mechanical properties	Porosity	Pore size	Type of cultured cells	Medium used	Maximum growth rate	References
Into hydrogel form	N/A	2.99%	4 to 6 µm	HeLa cells (immortalized human cervical cancer cells)	Dulbecco’s modified eagle’s medium (DMEM)	Cell viability of 95%	[51]
N/A	1.26 ± 0.16 MPa	close to 90%	102.7 ± 39.9 µm	Mesothelial Cells and Mesothelium Tissue	Dulbecco’s Modified Eagles Medium (DMEM) and fetal bovine serum (FBS)	DNA content of cells: 1.38 µg	[52]
Ethylene dichloride (EDC)/N-hydroxysuccinimide (NHS)	N/A	N/A	N/A	Cornea epithelial cells	Dulbecco’s Modified Eagles Medium	Cell number: 2.8 × 10^4^ to 3.8 × 10^4^ cells	[53]
EDC/NHS	4 MPa	N/A	N/A	Endothelial cells and smooth muscle cells	Fetal calf serum (FCS) and Minimum Essential Medium	Increased significantly in HE staining results	[54]

**Figure 4.**
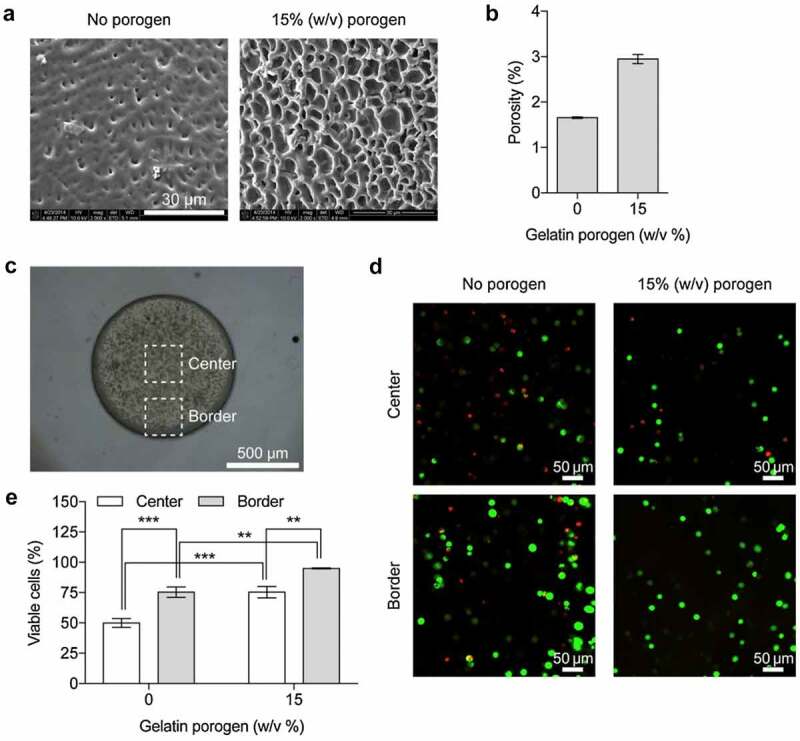
3D culture in porous structures of gelatin-methacrylate hydrogel scaffolds. (a) Representative scanning electron micrographs showing porous structures of gelatin-methacrylate hydrogel scaffolds fabricated with no porogen (left) and 15% (w/v) of gelatin porogen (right). (b) Bar graph presenting porosity measured in the gelatin-methacrylate scaffolds shown in (A). Error bars represent standard deviation. (c) Optical micrograph showing a HeLa cell-seeded gelatin-methacrylate scaffold at seeding density of 5 × 10^6^ cell/ml. White dotted squares (center and border) indicate regions of interest for estimation of cell viability as presented in (d) and (e). (D) Fluorescence micrographs showing live (green) and dead (red) cells at the center (top) and border (bottom) in gelatin-methacrylate scaffolds fabricated with no porogen (left) and 15% (w/v) of gelatin porogen (right). (E) Bar graph displaying cell viability at the center (white) and border (gray) in gelatin-methacrylate scaffolds fabricated with no porogen and 15% (w/v) of gelatin porogen [51].

The porosity of gelatin methacrylate scaffolds was manipulated through porogen leaching during cross-linking under UV irradiation [51]. The gelatin methacrylate was prepared from gelatin solution (15% (w/v)) and metacrlic anhydride. The photoinitiator, 2-hydroxy-1-(4-(hydroxyethoxy)phenyl)-2-methyl-1-propanone) and the porogen, either gelatin or polyethylene glycol were blended with the gelatin methacrylate solution. The mixture was subsequently poured into polydimethylsiloxane (PDMS) microfluid channel, followed by cross-linking (10–30 s) and porogen leaching to form microculture scaffolds. Porosity increased significantly with porogen amount, especially polyethylene glycol. However, a large pore size around 4 to 6 μm formed when 15% (w/v) of gelatin was used as the porogen. During the cultivation of the immortalized human cervical cancer cells (HeLa cells), more oxygen and nutrients could be delivered through the more porous scaffold. Hence, cell viability was increased up to 95%. The 3D culture in porous structures of gelatine-methacrylate hydrogel scaffolds is shown in Figure 4. Macroporous scaffolds were fabricated from 5% gelatin in 2-(N-morpholino) ethanesulfonic acid (MES) buffer containing EDC or 5% gelatin with 0.25% hyaluronic acid in MES buffer through cryogelation at −17°C for 1 min [52]. After incubation in water for 15 h for the completion of cross-linking. Hyaluronic acid as low as 5% could induce fast degradation and elevate the elastic modules. The scaffold properties such as pore size, porosity, water uptake, swelling were not affected, although the ultimate compressive stress (strain) and toughness were reduced. During the in vitro cell culture, the viability of rat mesothelial cells was proven by the DNA content up to 1.4 µg in gelatine scaffold, and 1.2 µg in gelatin/hyaluronic acid scaffold. The is due to the involvement of autocrine growth factor in mesothelial cells was up-regulated. Gelatin/chitosan scaffolds were synthesized using EDC/NHS crosslinkiers for the corneal epithelial cells implantation [53]. The crosslinkers were dissolved in ethanol solution (95%) and added to the mixture of gelatin/chitosan dried in the oven at 40°C for 1 d. Subsequently, the cross-linked scaffold was frozen at −20°C for 7 h and −80°C for 1 d. The gelatin/chitosan wet scaffolds with a composition ratio of 30/70 showed high transparency of 75% and water content to suit the human cornea. The gelatin/chitosan with the composition ratio of 2:8 exhibited the least degradation. The SEM micrographs and optical microscope images showed the proportion of the cells suspension attached up to 73.8% and 68.2% in the gelatin/chitosan scaffolds with the composition ratio of 2:8 and 3:7, respectively. The use of chitosan did not result in immune responses against transplant target, signifying that the gelatin/chitosan scaffolds could be a biocompatible hydrogel in corneal tissue engineering. In addition, gelatin was added in coaxial fibers vascular membranes and crosslinked with EDC/NHS for the production of artificial blood vessels [54]. The mechanical properties were improved, and the burst pressure reached up to 2844.55 ± 272.65 mmHg after crosslinking. Hence, the number of cells increased significantly in HE staining results.

### 2.5 Cellulosic culture scaffolds

Cellulose is a polysaccharide that serves as the building block of the plant cell wall. It is abundantly available for different biomedical purposes. A recent review revealed that decellularized plants had been extensively studied in the 3D cell culture [55]. Another review on genetic modification to enhance bacterial cellulose production and its application to be used as scaffold material showed that its excellent properties in tissue engineering [56]. In addition, many cellulose derivatives were commercially produced, including cellulose acetate with excellent flexibility and mechanical properties to form robust culture scaffolds.

Bacterial nanocellulose (BNC) with nanofibers produced by several bacterias such as *Gluconacetobacter xylinus* can be used as the culture scaffold as well. The nanocellulose isolated from cellulose, namely cellulose nanofiber (CNF) and cellulose nanocrystal (CNC), receive equal attention to BNC as they possess remarkable mechanical properties. The cellulosic culture scaffolds except the decellularized plant culture scaffolds are summarized in Table 7. Cellulose culture scaffolds were mainly used in the cultivation of epidermal cells for wound healing. The antibacterial properties of cellulose inhibited the growth of bacteria in the cultivation thus contribute additional properties to the culture scaffold.

**Table 7.** Cellulosic culture scaffold

Type of cellulose	Mechanical properties	Porosity	Pore size	Type of cultured cells	Medium used	Maximum growth rate	References
Bacterial cellulose	Tensile strength: 157.90 ± 23 MPa	N/A	N/A	Epidermal cells cultivation	Dulbecco’s modified Eagle’s medium	Cell area: 83.3 µm^2^	[57]
Cellulose acetate	Elongation at break: 247.00 ± 47.27%	91.83 ± 14.62%	N/A	Bacteria cells *S. aureus, S. epidermidis*, and *E. coli*	Nutrient agar	Reduced bacteria inhibition	[58]
Bacterial cellulose	N/A	N/A	N/A	Human dermal fibroblast cultivation	Dulbecco’s modified Eagle’s medium	Antibacterial rates: 96.6 ± 0.5% against *S. aureus* and 90.6 ± 1.6% against *E. coli*.	[60]
Bacterial cellulose	N/A	N/A	N/A	U251 Human glioblastoma cancer cell line	Dulbecco’s modified Eagle’s medium	Cell viability: 110%	[59]

Polysaccharides scaffolds made from cellulose and/or chitin were coated with BC or collagen nanofibers using a spin coater [57]. The cellulose scaffolds with 5 layers of collagen nanofibers coating recorded the highest tensile strength of 157.90 ± 23 MPa while cellulose/chitin scaffold with 5 layers of BC nanofibers coating attained a tensile strength of 135.49 ± 17 MPa. The surface wettability of scaffolds was greatly improved by BC nanofibers coating, showing water contact angles in the range of 56.55–83.19 °. The collagen nanofibers coatings resulted in a hydrophobic surface with water contact angles within 90.06–102.17 ° were recorded. The scaffolds coated with BC nanofibers did not induce any cell proliferation on its surface. However, the scaffolds with 3 layers of collagen nanofibers coating allowed cell attachment and proliferation during the cultivation of epithermal cells.

Furthermore, casting and phase inversion methods were combined to develop PVA/CA thin films for wound healing [58]. PVA was dissolved in dimethylformamide (DMF) at 130°C, then cellulose acetate was added to the PVA/DMF mixture. The mixture was then cast into a thin film and immersed into a salt solution containing 8 wt% of Na_2_SO_4_ and 4 wt% of NaOH. The highest mechanical resistance was recorded from PVA/CA film prepared at the ratio of 90:10 with the breaking strength of 2.56 ± 0.14 N and the elongation at break of 247.00 ± 47.27%. The increasing PVA content promoted the flexibility degree of PVA/CA thin film. All PVA/CA films showed a mass degradation of about 20% after being incubated in different physiological media for 28 d. The antimicrobial properties of the films were tested with *Staphylococcus aureus, Staphylococcus epidermidis*, and *Escherichia coli*. The antibacterial properties were improved by adding antimicrobial peptide LL37, but independent from the film composition. The albumin interface reduced the bacteria inhibition zone in all PVA/CA films. The protein adhered to the antimicrobial agents reduced of free fraction for inhibition.

**Figure 5.**
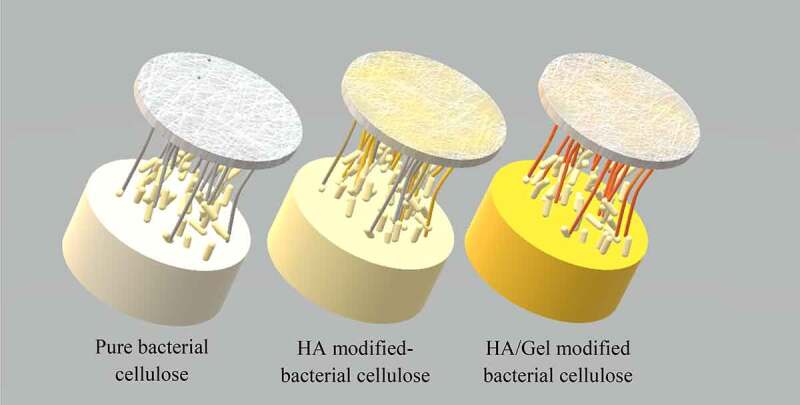
Schematic diagram of BC based scaffolds [59].

BNC scaffold was modified with hyaluronic acid and gelatin through *in situ* fermentation for the application in glioblastoma cell culture [59]. *Gluconacetobacter xylinus* was cultivated on the Hestrin & Scharamm medium containing hyaluronic acid and/or gelatin for 10 d at 28°C to produce the composite scaffolds. The schematic diagram of BC based scaffolds is shown in Figure 5. Human glioblastoma cancer cell line U251 was then cultured into BNC, BNC/hyaluronic acid, and BNC/hyaluronic acid/gelatin composite scaffolds. NBC/HA/Gel composite scaffold with 10 mg/ml of gelatin showed a moderate hydrophilic surface with a water contact angle of 71.23 ± 5.39 °, which could support cell adhesion and proliferation. Besides that, the cell proliferation on BC/hyaluronic acid/gelatin composite scaffolds raised from 75% to 135% cell viability after 7 d compared to BNC and BNC/hyaluronic acid culture scaffolds. Moreover, the cells cultivated on BNC/hyaluronic acid/gelatin scaffolds exhibited better vitality and formed multilayered growth and dense cell clusters.

Other than that, BNC membranes were modified using 3-aminopropyltriethoxysilane (APTES) in ethanol (0.125–0.5 vt%) to create antibacterial properties for biomedical applications [60]. The wettability of the BC membrane reduced with the water contact angle of 47.6 ± 8.7 ° increased 84.3 ± 8.0 ° when 0.5 vt% of APTES was used in the modification. The increased surface hydrophobicity improved the antibacterial properties of the membrane. BNC membrane with 0.5 vt% of APTES showed an antibacterial rate of 96.6 ± 0.5% against *S. aureus* and 90.6 ± 1.6% against *E. coli*. The cell viability was not significantly affected by the APTES concentration used in the modification. Hence, APTES modified BNC membranes were claimed to be nontoxic when applied in the cultivation of normal human dermal fibroblasts.

### 2.6 Agarose culture scaffold

Agarose is a polysaccharide extracted from agar or red seaweeds. It can be converted into porous gel through thermal treatment of agarose in a buffer besides working as a gelling agent applicable in nucleic acid electrophoresis, immunodiffusion, chromatography, and more. Table 8 shows the agarose culture scaffolds developed recently. Agarose was blended with peptides such as aniline tetramer and laminin peptides to enhance cell attachment and cell proliferation, as reported in the literature. It was widely used in wound healing as it showed encouraging results in the cultivation of epidermal cells.

**Table 8.** Agarose culture scaffolds

Crosslinkers	Mechanical properties	Porosity	Pore size	Type of cultured cells	Medium used	Maximum growth rate	References
N/A	N/A	N/A	N/A	neuron cells for neuroregeneration	Dulbecco’s modified Eagle’s medium	Cell viability: 1.1	[61]
N/A	N/A	N/A	N/A	Corneal epithelial cells	Dulbecco modifid Eagle medium	Wound healing surface area: 25 mm^2^	[62]
N/A	N/A	N/A	N/A	Human dermal fibroblasts	Dulbecco’s modified Eagle’s medium	Cell growth rate:182% at day 7	[63]

Agarose/alginate-aniline tetramer scaffold was synthesized with varying aniline tetramer content [61]. Alginate Di-Aldehyde was first synthesized to allow the attachment of aniline tetramer. The attachment stabilized the incorporation of aniline tetramer in agarose. The hydrogel could be electrically controlled to release a drug for neuroregeneration. The hydrogel with 10% aniline tetramer showed the maximum ionic conductivity of 4.312 × 10^−11^ S/cm compared to the ionic conductivity of neat agarose hydrogel, which is 2.025 × 10^−11^ S·cm^−1^. The ionic conductivity increased due to the higher number of ions generated by aniline tetramer. The amount of released dexamethasone increased from 1.2 to 9.3% with the increase of the amplitude of electrical voltage from 1 V to 3 V. The hydrogel with 10% of aniline tetramer also exhibited the highest cell viability at day 5 with no cytotoxicity to the culture representing a potential substrate for cell growth. An agarose-based dome scaffold was developed to heal dog and rabbit corneal wounds in dogs and rabbits [62]. The cornea epithelial cells were cultured on an agarose scaffold for 21 d, and the surface area of corneal fluorescein retention was measured every 6 or 12 h until full corneal epithelialization was detected. Dome scaffolds were fabricated using the polyvinylsiloxane concave template on 3D printed acrylonitrile butadiene styrene cylinder support. Agarose mixture with the maintenance medium was used to fill the corneas. All dog and rabbit corneas and rabbit corneas showed a circular, diffuse, cloud-like pattern of optical haze on the dome scaffolds. The dog corneas cultured on flat scaffolds showed a focal and crater-like region of optical haze. More importantly, all corneas on the agarose scaffolds maintained convex curvature throughout the cultivation period. Agarose scaffolds was functionalized with aldehyde via (2,2,6,6-tetramethylpiperidin-1-yl)oxyl (TEMPO) oxidation [63]. They further functionalized aldehyde/agarose scaffolds with laminin peptides, namely CGG-A99 and CGG-AG73. The peptides were incorporated through thiazolidine formation without coupling reagents. In addition, peptide-agarose microgels were produced by dropping the peptide-agarose gel into acetate buffer at pH 5 with the subsequent addition of laminin peptides for 2 h. The microgel formed after cysteine quenching. During 2D cell cultivation of human dermal fibroblasts, the peptide-agarose gels allowed cell adhesion up to 120% and enhanced cell proliferation about 180%. The peptide-agarose microgels supported 3D cultivation of human dermal fibroblasts and helped the cell to spread and proliferate.

### 2.7 Alginate culture scaffold

Alginate is an anionic polymer derived from brown seaweed. It has been extensively used for different biomedical purposes, including dressing for wound healing. Alginate culture scaffolds were often blended with additives to enhance their mechanical strength. The cell viability also increased when alginate culture scaffolds were synthesized in the form of beads.

Growth plate chondrocytes of the neonatal mouse were confined within alginate hydrogel beads [64]. Sodium alginate in phosphate-buffered saline (1.5% (w/v)) was dripped together with growth plate chondrocytes through the pipette tip into the mixture of CaCl_2_ and NaCl solution. Using the bead culture scaffolds, the confined chondrocytes showed high viability greater than 90%, cartilage matrix deposition between 15 to 20%, low hypertrophy level with only 0.25 fold change, and a continuous rise in cell proliferation up to 20% over 7 days in culture. Further modified the alginate bead cultures with thyroxine created a discrete domain of hypertrophic cells that have a similar structure to the native growth plate cartilage.

## 3. Conclusions and future perspective

In general, the biopolymer culture scaffolds enhanced cell proliferation and differentiation significantly compared to the 2D culture mediums. The cultivation under dynamic conditions further promoted oxygen and nutrient diffusion. The critical findings on different biopolymer culture scaffolds are summarized as follows.

- Collagen culture scaffolds were mainly synthesized through freeze-drying and ice templating to form aligned and uniform pores. Although crosslinkers (EDC and NHS) increased the scaffold stability, they weakened the mechanical strength. By increasing the collagen concentration in the scaffold, porosity and pore sizes could be enlarged.
- Chitosan was commonly dissolved in acetic acid for preparing culture scaffolds. Different additives such as peptides, calcium phosphate, and Au were added to increase the cell adhesion and proliferation besides improving the mechanical properties of chitosan scaffolds. The chitosan scaffolds showed encouraging results after being extensively tested in the cultivation of mesenchymal stem cells.
- Silk fibroin culture scaffolds were commonly incorporated with chitosan to increase their hydrophilicity, porosity, and pore size through the ice-templating method. The silk fibroin culture scaffolds were also extensively tested in the cultivation of mesenchymal stem cells. The thermal stability and mechanical strength of the silk fibroin scaffolds were enhanced when reduced graphene and chondroitin were incorporated.
- Gelatine culture scaffolds were frequently used for the cultivation of epithelial cells and fibroblast cells. The porous structure formed in the gelation culture scaffolds allowed more oxygen and nutrient to diffuse into the cells for increasing cell viability.
- Cellulose culture and agarose scaffolds were often applied in the cultivation of epidermal cells for wound healing. Agarose culture scaffolds were further blended with peptides to enhance cell attachment and cell proliferation.
- The alginate culture scaffold required other additives to enhance its mechanical strength. The cell viability was increased using alginate culture scaffolds in the form of beads.

Future studies should include more integration of culture scaffold into different bioreactors for large-scale production. The nutrient flow rate and the oxygen diffusion rate in a bioreactor can be regulated to increase the cell viability throughout the system and avoid necrotic cores with insufficient ECM. The porous scaffolds can be integrated with different bioreactors, including spinner flasks, rotating wall vessels, perfusion bioreactors, Quasi Vivo systems, and microfluidic systems devices to enhance cell cultivation. Besides flat culture scaffolds, culture scaffolds in varied forms including fibers, hollow fibers, beads, and more to ease the scaffold placement and removal for cell cultivation and post-analysis [65]. Culture scaffolds are commonly fixed at the container cover of a spinner flask and threaded in needles for bone tissue engineering [66]. ECM production is only permitted on scaffold surface. Turbulent shear due to media mixing affects cell growth and tissue formation. In addition, culture scaffolds are rotated insides the rotating wall vessels filled with culture medium and rotated along a horizontal axis. Cell lines can be cultured on the scaffold, but frequent collisions result in low cell density [67]. In perfusion bioreactors, the medium flows through or over a cell population on culture scaffolds. The penetration of oxygen and nutrients is promoted through the pores of culture scaffolds [68]. Incorporating culture scaffolds in the microfluidic system with a slim volume for seeding and reagents remains challenging, and it needs a more creative solution [69].

Looking into the Sustainable Development Goals (SDGs), biopolymeric culture scaffolds play an essential role in SDG 3 Good Health and Well-Being as they enable biomedical studies at low cost, involving tumor cells, wound healing, and more. Therefore, the aim to reduce the global mortality rate by the year 2030 is achievable even for the bottom billions. Moreover, biopolymers are green materials that help to achieve SDG 13 Climate Action. It helps to reduce the use of nonrenewable and non-biodegradable materials, reducing the carbon footprint of biomedical industry.

## Highlights

- Natural biopolymeric culture scaffold enhanced cell growth.
- Natural biopolymers intrinsically have higher hydrophilicity and porosity.
- Porous structure of biopolymeric scaffold allowed efficient air diffusion for cells.
- Different biopolymer scaffolds are suited for different types of cell cultivation.
